# Combined Analysis of the Fruit Metabolome and Transcriptome Reveals Candidate Genes Involved in Flavonoid Biosynthesis in *Actinidia arguta*

**DOI:** 10.3390/ijms19051471

**Published:** 2018-05-15

**Authors:** Yukuo Li, Jinbao Fang, Xiujuan Qi, Miaomiao Lin, Yunpeng Zhong, Leiming Sun, Wen Cui

**Affiliations:** Zhengzhou Fruit Research Institute, Chinese Academy of Agricultural Sciences, Zhengzhou 450009, China; liyukuokiwi@126.com (Y.L.); linmiaomiao@caas.cn (M.L.); zhongyp_126@126.com (Y.Z.); sleiming@163.com (L.S.); cuiwenky@126.com (W.C.)

**Keywords:** candidate genes, metabolites, flavonoid biosynthesis, fruit coloring, *Actinidia arguta*

## Abstract

To assess the interrelation between the change of metabolites and the change of fruit color, we performed a combined metabolome and transcriptome analysis of the flesh in two different *Actinidia arguta* cultivars: “HB” (“Hongbaoshixing”) and “YF” (“Yongfengyihao”) at two different fruit developmental stages: 70d (days after full bloom) and 100d (days after full bloom). Metabolite and transcript profiling was obtained by ultra-performance liquid chromatography quadrupole time-of-flight tandem mass spectrometer and high-throughput RNA sequencing, respectively. The identification and quantification results of metabolites showed that a total of 28,837 metabolites had been obtained, of which 13,715 were annotated. In comparison of HB100 vs. HB70, 41 metabolites were identified as being flavonoids, 7 of which, with significant difference, were identified as bracteatin, luteolin, dihydromyricetin, cyanidin, pelargonidin, delphinidin and (−)-epigallocatechin. Association analysis between metabolome and transcriptome revealed that there were two metabolic pathways presenting significant differences during fruit development, one of which was flavonoid biosynthesis, in which 14 structural genes were selected to conduct expression analysis, as well as 5 transcription factor genes obtained by transcriptome analysis. RT-qPCR results and cluster analysis revealed that *AaF3H*, *AaLDOX*, *AaUFGT*, *AaMYB*, *AabHLH*, and *AaHB2* showed the best possibility of being candidate genes. A regulatory network of flavonoid biosynthesis was established to illustrate differentially expressed candidate genes involved in accumulation of metabolites with significant differences, inducing red coloring during fruit development. Such a regulatory network linking genes and flavonoids revealed a system involved in the pigmentation of all-red-fleshed and all-green-fleshed *A. arguta*, suggesting this conjunct analysis approach is not only useful in understanding the relationship between genotype and phenotype, but is also a powerful tool for providing more valuable information for breeding.

## 1. Introduction

Kiwifruit (*Actinidiaceae*, genus *Actinidia*), a kind of perennial and deciduous plant, is one of the fruit trees that have been domesticated and cultivated successfully in the last century, and in recent years has been grown commercially worldwide [1,2]. As a fruit tree originating from China, the genus *Actinidia* comprises a total of 76 species and about 125 known taxa worldwide [3], but among these, only two species—*Actinidia chinensis* and *A. deliciosa*—have been cultivated commercially [4].

Flesh color is an important quality characteristic and is also an important index for evaluation of fruit quality in *Actinida*. The two species mentioned above, *A. chinensis* and *A. deliciosa*, have dominated the international market because of their large fruit, high vitamin C content, unique flavor, and long storage period properties [5]. In addition, these two species are often used as experimental materials for the study of flesh coloring, which mainly concentrate on green- and yellow-fleshed coloring, corresponding to chlorophyll metabolism and carotenoid metabolism, respectively. In recent years, because of their rich functional components (phenols and flavonoids), red-fleshed kiwifruit has attracted more and more attention from consumers and researchers.

Flavonoids, secondary metabolites found in plants, contribute to plant environmental adaptation [6], fruit development [7,8], and even human health [9]. These compounds accumulate in various tissues at different developmental stages [10], and they are also the direct factors that cause red color in *Actinida* flesh [11,12,13]. Flavonoid biosynthesis controlled by a diverse array of exogenous and endogenous factors is the most studied secondary metabolic pathway in plants, including *Actinidia*. Most structural gene-coding enzymes have been studied in *Actinida* [11,14,15,16,17,18], as well as transcription factors [13,19]. However, most of the reports on red-fleshed *Actinidia* coloring are mainly concentrated on *Actinidia chinensis*, and rarely examine *Actinidia arguta*, which is a kind of all-red-fleshed mini-type *Actinidia*.

The post-genomic era is densely populated with databases reporting the results of “-omics” studies, which provide insights into genome-wide approaches on the basis of comprehensive analysis of transcriptome, proteome, and metabolome, in addition to the traditional molecular biological, biochemical and reverse-genetic approaches [20,21,22]. Accordingly, metabolomics is the attempt to identify and quantify all endogenous small molecule metabolites in an organism or biofluid sample [23]. Furthermore, metabolomics analysis can be regarded as a technical means of association analysis together with other data to analyze the gene function involved in the metabolic pathway of interest and can also provide supporting information for gene mining. In recent years, combined high-throughput methods have been used to study color development. Combined metabolome and transcriptome analyses in fruits have clarified the relationship between the contents of various secondary metabolites and the corresponding differentially expressed genes, broadening the global view of plant color regulation [24,25,26].

To investigate the metabolite changing rule associated with color change during *Actinidia* development, two different cultivars—“Hongbaoshixing” (“HB”) and “Yongfengyihao” (“YF”)—were selected to perform metabolome and transcriptome analysis. All metabolites that could be detected by instrument were identified and then used for differential analysis. Finally, 41 metabolites related to flavonoid biosynthesis were obtained, 7 of which presented significant difference in the comparison between HB100 and HB70. Through combined metobolome and transcriptome analysis, 2 pathways—flavonoid biosynthesis and galactose metabolism—were significantly different in terms of change during fruit development. In flavonoid biosyntheis, 8 differentially expressed structural genes were obtained. In addition, 7 transcription factor genes related to flavonoid biosynthesis were obtained by transcriptome analysis. RT-qPCR results and cluster analysis revealed the genes *AaF3H*, *AaLDOX*, *AaUFGT*, *AaMYB*, *AabHLH* and *AaHB2* could be determined as candidate genes on which to conduct further study. Through integrated analysis of identified metabolites with significant difference and related genes involved in flavonoid biosynthesis, the association between metabolites and genes was explained so as to provide valuable information for revealing the red coloring of *A. arguta*.

## 2. Results

### 2.1. Metabolite Identification

**Figure 1:**
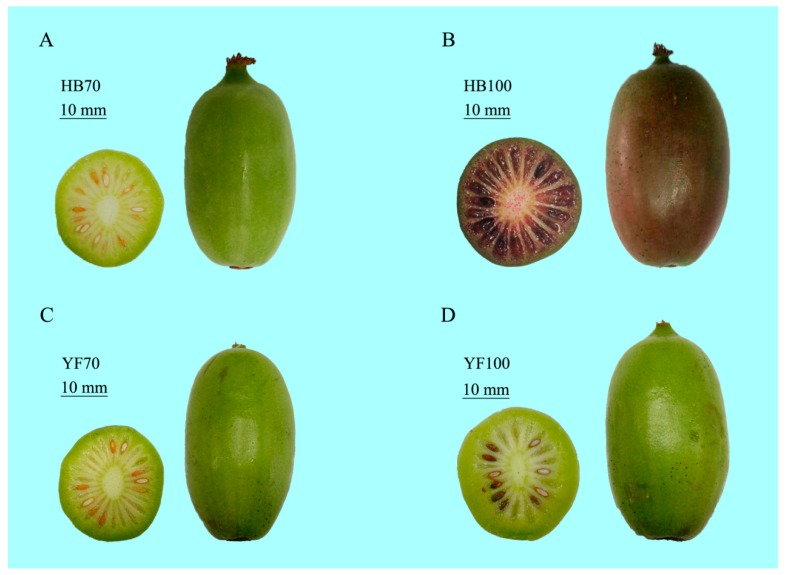
The phenotype of *Actinidia arguta* cv. “HB” and “YF” at two different sampling stages 70 DAFB and 100 DAFB, respectively. (**A**) Green fruit “HB” at 70 DAFB, HB70; (**B**) Red fruit “HB” at 100 DAFB, HB100; (**C**) Green fruit “YF” at 70 DAFB, YF70; (**D**) Green fruit “YF” at 100 DAFB, YF100.

In order to explore the change of metabolites during *A. arguta* fruit development (Figure 1), a metabolome program was carried out. Only the m/z and chromatographic retention time were collected for each detected feature. Therefore, the quality control for features obtained by XCMS software was conducted by the overall MS signal intensity controlled by TIC (total ion chromatogram) (Figure S1), evaluation of width for m/z peak, and assessment of width for retention time peak for each feature (Figure S2).

The original wiff format data was converted to the mzXML format using MSConvert software [27], followed by alignment and extraction of the peak and calculation of the peak area. The final statistics showed that 18,598 and 10,239 metabolites were obtained by the POS (positive) and NEG (negative) models, respectively, 9016 and 4699 of which were annotated (Table 1).

**Figure 2:**
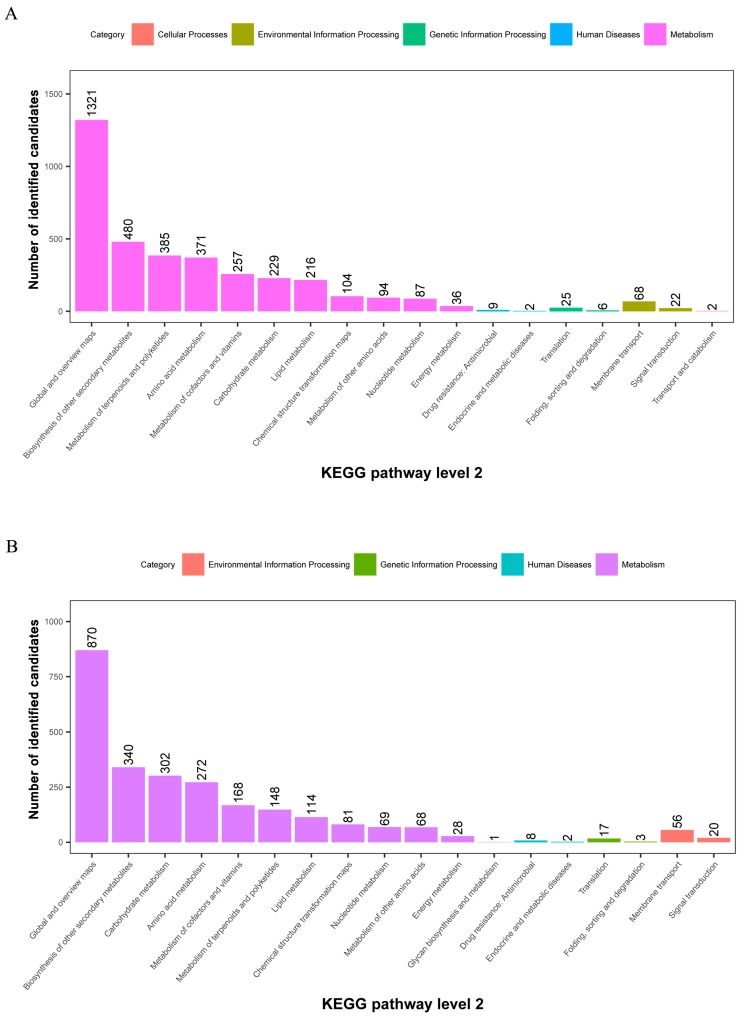
KEGG pathway classification: metabolites detected and annotated. (**A**) POS model; (**B**) NEG model. The *x*-axis represents level-2 terms of the KEGG pathway and the *y*-axis represents the number of metabolites identified.

**Figure 3:**
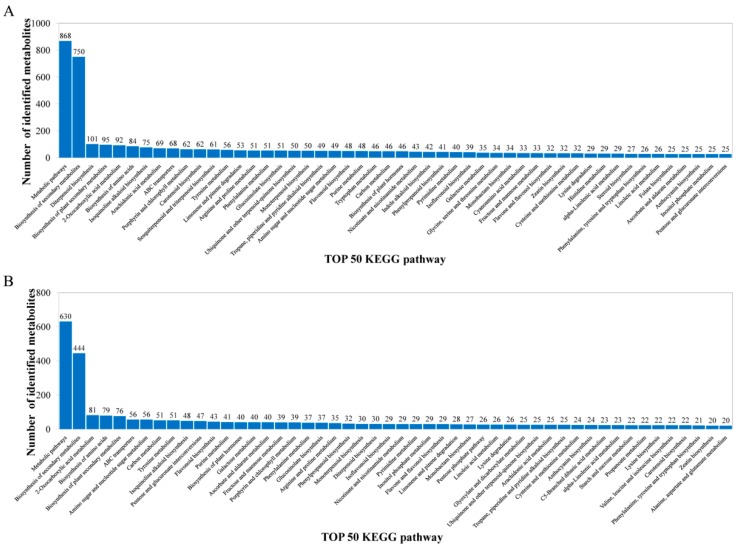
Identified metabolites classified into the top 50 KEGG pathways. (**A**) POS model; (**B**) NEG model. The *x*-axis represents the top 50 KEGG pathways and the *y*-axis represents number of identified metabolites involved in this pathway.

The metabolites identified above were assigned to the KEGG and PLANTCYC databases. All 8070 and 4321 metabolites were classified into 18 KEGG second-grade pathways, 3580 and 2461 of which were classified into “metabolism” in the POS and NEG models, respectively. For the “metabolism” term, the top priority was “Global and overview maps”, followed by “biosynthesis of other secondary metabolites”, ”metabolism of terpenoids and polyketides”, “amino acid metabolism”, “metabolism of cofactors and vitamins” and “carbohydrate metabolism” (Figure 2). 480 and 340 metabolites were assigned to “biosynthesis of other secondary metabolites” in the POS and NEG models, respectively. Of these, 73 and 67 metabolites were involved in “flavonoid biosynthesis” and “anthocyanin biosynthesis” in the POS and NEG models, respectively (Figure 3).

**Figure 4:**
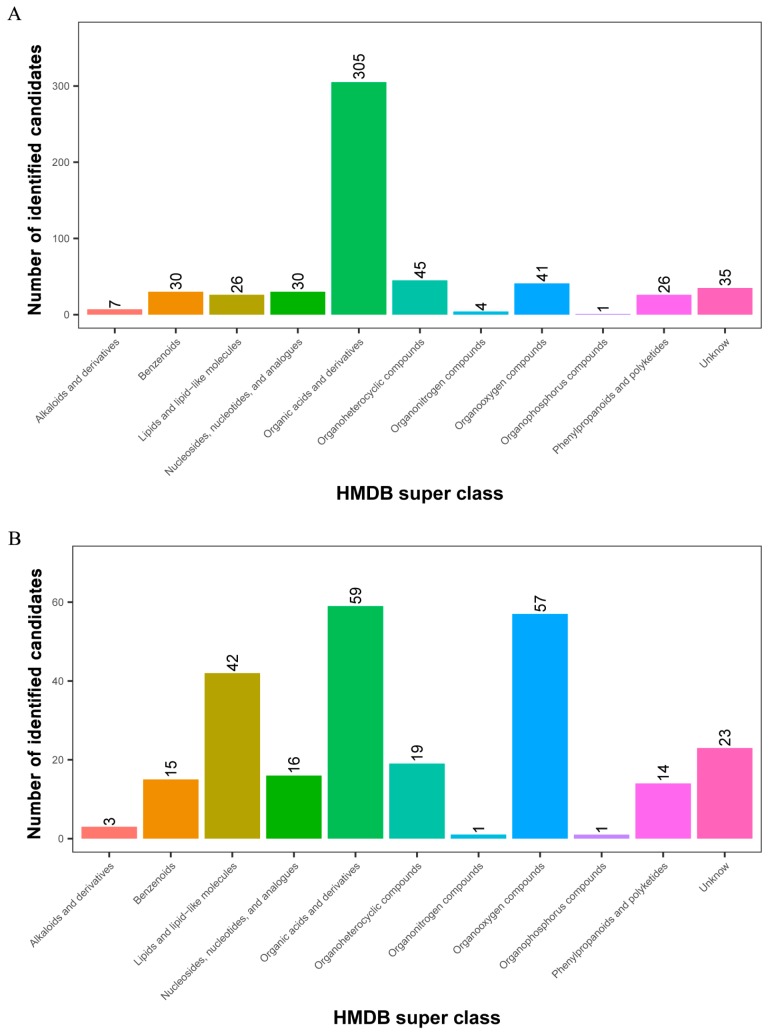
Identified metabolites from level-two identification were classified into 11 HMDB super classes. (**A**) POS model; (**B**) NEG model. The *x*-axis represents HMDB super classes, and the *y*-axis represents the number of identified metabolites.

All 1267 and 360 metabolites obtained from level-two identification were assigned to the HMDB database, 550 and 250 of which were matched and classified into 11 HMDB super classes in POS and NEG, respectively. Of these, 305 metabolites were included in the “organic acids and derivatives” term, which presented the majority of all classes in the POS model. Meanwhile, “organic acids and derivatives”, which contained 59 metabolites, was the first class, followed by “organooxygen compounds” and “lipids and lipid-like molecules” in the NEG model (Figure 4).

### 2.2. Identified Metabolites Involved in Flavonoid Biosynthesis

In order to obtain quantitative information on the metabolites, 14,132 and 8400 high-quality metabolites obtained from the 18,598 and 10,239 of all metabolites were used for differential analysis (Table 2). According to the quantitative results of the identified metabolites, the differential metabolites between different group comparisons were analyzed based on fold-change and p-value. In the comparison between HB100 and HB70, a total of 2421 and 1838 metabolites presented as being up-regulated and down-regulated, respectively (Table 3). In addition, a total of 41 metabolites were identified as being flavonoids among the high-quality metabolites, 7 of which were with significant difference, and were identified as bracteatin, luteolin, dihydromyricetin, cyanidin, pelargonidin, delphinidin and (−)-epigallocatechin (Table S1 and Table 4).

### 2.3. Analysis of Transcription Factors

**Figure 5:**
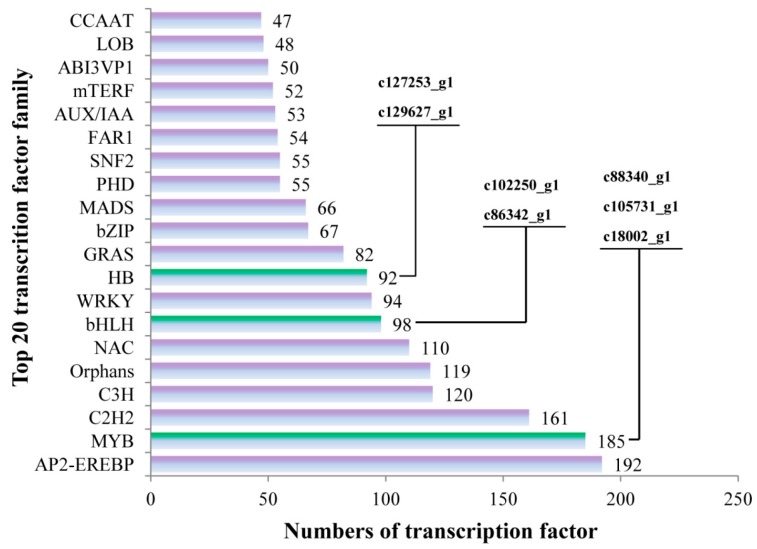
The numbers of transcription factors involved in the top 20 transcription factor families. The *x*-axis represents the numbers of transcription factors and the y-axis represents the top 20 transcription factor families.

A total of 2614 transcription factor genes were identified through transcriptome data analysis and could be grouped into 80 transcription factor families (Table S2), the majority of which were “AP2-EREBP”, “MYB” and “C2H2”, followed by “C3H“, ”Orphans“, “NAC”, “bHLH”, “WRKY” and “HB”. Of these 2614 transcription factor genes, 7 were annotated as being associated with flavonoid biosynthesis. The “c88340_g1”, “c105731_g1 (*AaMYBC1*)” and “c18002_g1 (named *AaMYB*)”, “c102250_g1” and “c86342_g1 (named *AabHLH*)”, and “c127253_g1 (named *AaHB1*)” and “c129627_g1 (named *AaHB2*)” belonged to “MYB”, ”bHLH“ and “HB” families, respectively (Figure 5).

### 2.4. Comprehensive Analysis of Metabolome and Transcriptome

**Figure 6:**
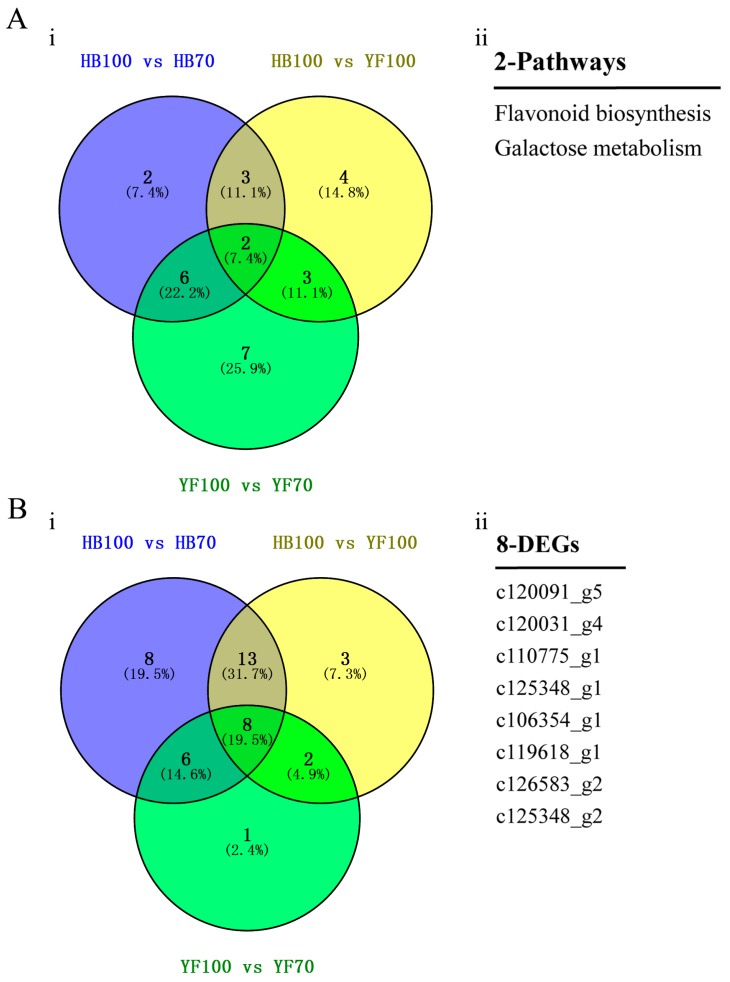
Identification of candidate DEGs. (**A**) Venn Diagram of pathways. (**i**) indicates Venn Diagram result between three groups, HB100 vs. HB70, HB100 vs. YF100, and YF100 vs. YF70. (**ii**) indicates the two specific pathways; (**B**) Venn Diagram of DEGs involved in flavonoid biosynthesis. (**i**) indicates Venn Diagram result between three groups, HB100 vs. HB70, HB100 vs. YF100, and YF100 vs. YF70. (**ii**) indicates specific 8 DEGs.

In order to investigate the association between metabolites and genes involved in the same biological process (KEGG Pathway), the comprehensive analysis of metabolome and transcriptome was performed using Pearson’s Correlation Coefficient [28,29]. The results showed that 646, 156 and 484 differentially expressed genes participated in 13, 18 and 12 pathways among three groups HB100 vs. HB70, HB100 vs. YF10 and YF100 vs. YF70, respectively (Table S3). The 2 pathways, “flavonoid biosynthesis” and “galactose metabolism”, were a common presence among the three group comparisons. In flavonoid biosynthesis, a total of 8 candidate DEGs were found (Figure 6).

### 2.5. RT-qPCR, Cluster and Phylogenetic Analysis

**Figure 7:**
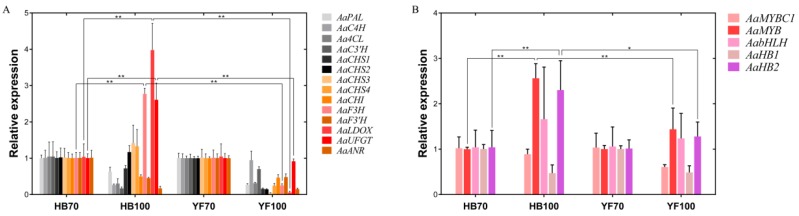
Expression profiles of structural and regulatory genes involved in flavonoid biosynthesis in the flesh of “HB” and “YF”. (**A**) 14 structural genes; (**B**) 5 regulatory genes. The *x*-axis represents two different development stages of “HB” and “YF”, and the *y*-axis represents relative expression. Data were analyzed using the *t*-test. * *p* < 0.05, ** *p* < 0.01.

**Figure 8:**
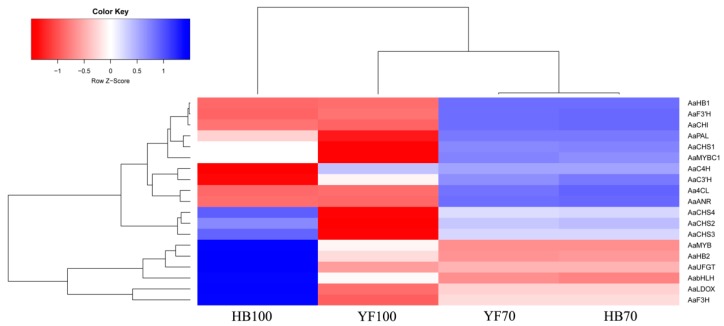
Cluster analysis for structural and regulatory genes. Blue boxes indicate high expression levels and red boxed indicate low expression levels.

**Figure 9:**
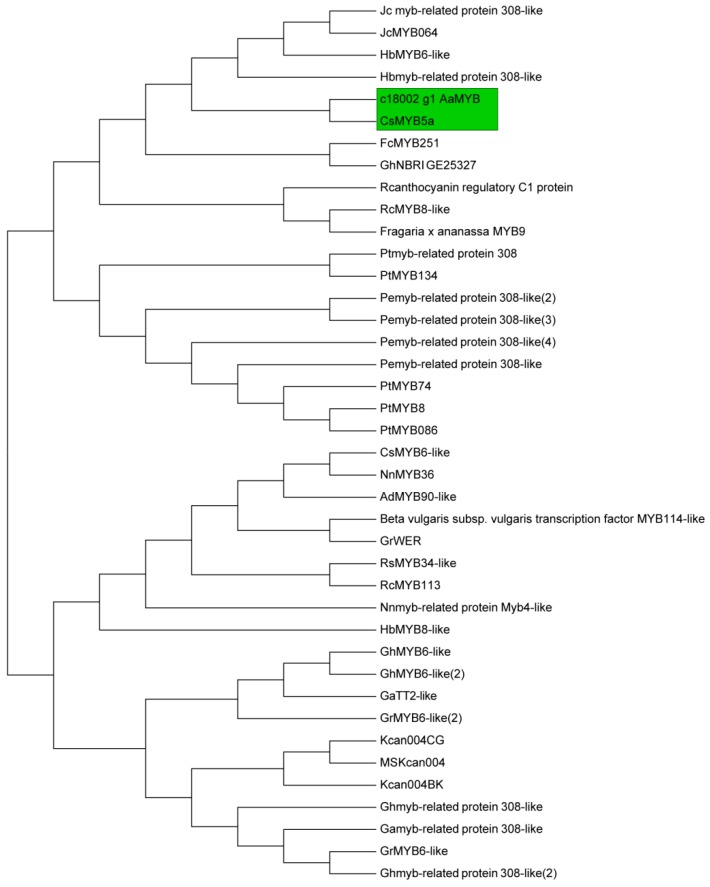
Phylogenetic analysis of 40 MYB transcription factor genes in different species. The green frame indicates that the two genes *AaMYB* and *CsMYB5a* are clustered together.

A total of 14 structural genes comprising the 8 DEGs found above and 6 other structural genes involved in flavonoid biosynthesis were determined to conduct RT-qPCR, as well as 5 regulatory genes *AaMYBC1*, *AaMYB*, *AabHLH*, *AaHB1* and *AaHB2* (Figure 7). The expression patterns of these three structural genes *AaF3H*, *AaLDOX* and *AaUFGT* were similar to the two regulatory genes *AaMYB* and *AaHB2*. The expression levels of *AaF3H*, *AaLDOX* and *AaUFGT* in “HB100” was significantly higher than those in “HB70” and “YF100” (Figure 7A). A similar rule of expression was detected for *AaMYB* and *AaHB2*. The expression levels of *AaMYB* and *AaHB2* in “HB100” were higher than those found in “HB70” and “YF100” (Figure 7B). Cluster analysis results showed that the genes *AaF3H*, *AaLDOX*, *AaUFGT*, *AaMYB*, *AabHLH* and *AaHB2* were clustered into the same class (Figure 8), suggesting that the expression patterns of these genes were similar to each other. Because MYB is the biggest and most important transcription factor family involved in flavonoids, *AaMYB* was used for BLAST in order to search for other *MYB* sequences in other species. A phylogenetic tree was constructed with the BLAST results. The results showed that *AaMYB* and *CsMYB5a* (*MYB5a* of *Camellia sinensis*) were clustered into one class (Figure 9).

### 2.6. Regulatory Network of Flavonoid Biosynthesis

**Figure 10:**
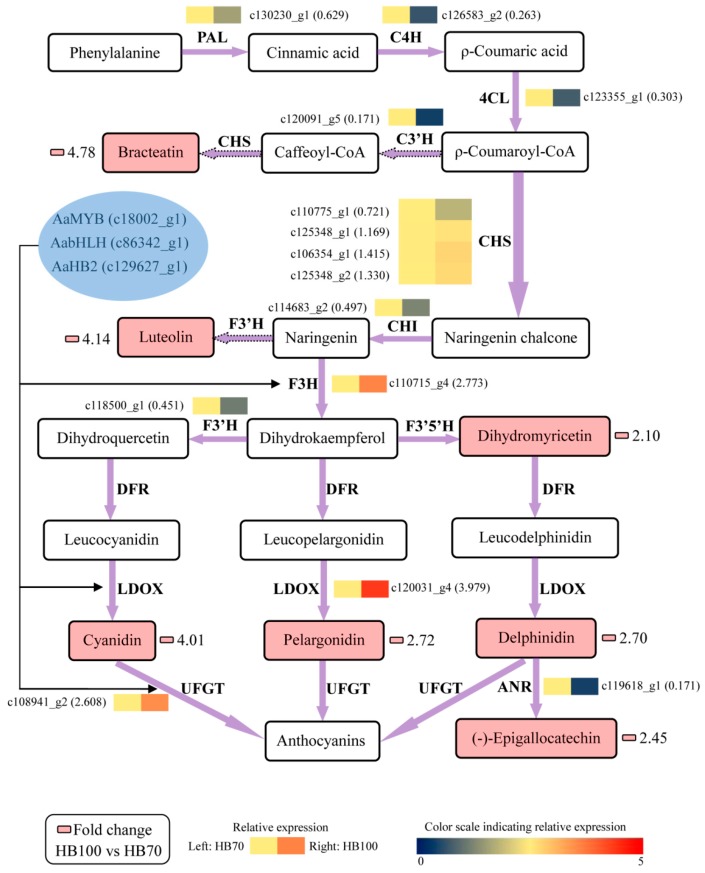
Regulatory network of flavonoid biosynthesis in two development stages “HB100” and “HB70”. HB100: “HB” kiwifruit at 100 DAFB; HB70: ”HB” kiwifruit at 70 DAFB. Color scale from dark blue to bright red represents relative expression levels 0–5. Small pink cells represent fold change of metabolites between HB100 and HB70. The solid frame arrow represents only one step of process, and the dotted frame arrow represents more than one step of process. PAL, phenylalanine ammonia-lyase; C4H, trans-cinnamate 4-hydroxylase; 4CL, 4-coumarate: CoA ligase; C3’H, coumaroylquinate 3’-monooxygenase; CHS, chalcone synthase; CHI, chalcone isomerase; F3H, flavanone 3-hydroxylase; F3’H, flavonoid 3’-hydroxylase; LDOX, leucoanthocyanidin dioxygenase; UFGT, UDP glucose-flavonoid 3-*O*-glcosyl-transferase; ANR, anthocyanidin reductase.

In order to better understand the relationship between metabolites and genes in flavonoid biosynthesis, all results of metabolites and genes were combined to establish a network, aiming to show the relationship between gene expression and metabolite accumulation more intuitively (Figure 10). Among the 14 structural genes, *AaF3H*, *AaLDOX* and *AaUFGT* were highly expressed in HB100 vs. HB70 in comparison with the other 11 structural genes. The 7 metabolites with significant differences in HB100 vs. HB70 of flavonoid biosynthesis were: bracteatin, luteolin, dihydromyricetin, cyanidin, pelargonidin, delphinidin and (−)-epigallocatechin. It is generally known that transcription factor plays a role by binding the promoter of structural genes. Therefore, we speculate the hypothesis that the three transcription factors AaMYB, AabHLH, and AaHB2 activate expression of *AaF3H*, *AaLDOX* and *AaUFGT* by binding the promoter of these three structural genes, inducing the accumulation of the 7 metabolites described above in flavonoid biosynthesis. Specifically, *AaMYB*, *AabHLH*, *AaHB2*, *AaF3H*, *AaLDOX* and *AaUFGT* were the candidate genes obtained from this study and could be used for further study, revealing the red mechanism of *A. arguta*.

## 3. Discussion

### 3.1. Metabolites Were Obtained by Metabolome Analysis

Plant metabolomics, a new field in the post-genome era [30], presents the rule of change of metabolites in various tissues. Metabolites are the final products of cell biological regulation process, and their level can be regarded as the response of plant development to genetic and environmental changes [31]. Therefore, metabolomic analysis can be used to investigate the relationship between biological processes and phenotypes; furthermore, some intuitive changes can also be observed at the metabolic level [32]. Up until now, several reports have examined the metabolic responses of different species to flavonoid biosynthesis, such as *Fagopyrum esculentum* [33], *Camellia sinensis* [34], and *Ficus carica L.* [35]. This research suggests that metabolome analysis plays a crucial role in explaining the molecular responses to flavonoid biosynthesis. In this study, through fruit metabolome analysis on two different *A. arguta* cultivars “HB” and “YF” at two developmental stages, a total of 28,837 metabolites were obtained, of which 13,715 had been annotated (Table 1). In addition, 22,532 high-quality metabolites were identified and selected for differential analysis (Table 2). The focus of our research was on flesh coloring, so the HB100 vs. HB70 comparison was selected as the object for further analysis. In the comparison HB100 vs. HB70, 41 metabolites were identified as being flavonoids, 7 of which, with significant difference, were identified as bracteatin, luteolin, dihydromyricetin, cyanidin, pelargonidin, delphinidin and (−)-epigallocatechin (Table S1). Such a result provides us with the insight that the difference between these 7 metabolites leads to the difference between red and green coloring in *A. arguta* flesh.

### 3.2. Transcription Factors Involved in Flavonoid Biosynthesis

Transcription factors are absolutely necessary for regulation of gene expression [36]. Normally, transcription factor proteins play a role through the combination of their own DNA-binding domain and the cis-acting element of their target genes [37,38]. In plants, transcription factors could participate in various biological process, including developmental regulation, defense elicitation, and stress responses [39,40,41,42,43]. Numerous studies have shown that flavonoid biosynthesis is regulated by the MBW complex, comprising MYB, bHLH, WD40 [44,45,46]. Therefore, finding transcription factors related to flavonoid biosynthesis is essential for investigating fruit coloring. In this study, 5 transcription factor genes were obtained by transcriptome analysis and used for expression analysis. The results showed that *AaMYB*, *AabHLH* and *AaHB2* were highly expressed at HB100, indicating that these three transcription factors might play a key role in fruit coloring in *A. arguta*. Phylogenetic analysis revealed that *AaMYB* was highly homologous to *CsMYB5a*, rather than the other *MYB* in *A. chinensis*, in which *AcMYB75* [47], *AcMYB110* [48] and *AcMYBF110* [13] were the key MYB transcription factors controlling fruit coloring, indicating that the MYB transcription factor that plays a key role in regulating flavonoid biosynthesis might be different in different *Actinidia* species.

### 3.3. Candidate Genes are Involved in Regulating Fruit Coloring

Genes, including structural and regulatory genes, involved in flavonoid biosynthesis and regulation have been found, studied and reported in many plants, including *Actinidia* [11,13,15,16,17,18,19]. However, most genes have been obtained and identified using traditional study technology. Since transcriptome analysis has come to be regarded as a crucial way to study the expression level, structure and function of genes in order to revealing phenotypic traits, combined analysis of transcriptome and metabolome has increasingly become a popular and practical tool for the mining of new genes involved in various metabolic pathways [49]. In this study, metabolome data, coupled with transcriptome profiling, was carried out in a combined analysis for the discovery of genes involved in flavonoid biosynthesis, thus searching for useful information to illustrate phenomenon of red coloring in *A. arguta* fruit. 19 genes, comprising 14 structural genes and 5 transcription factor genes, were obtained and used to analyze expression level; additionally, cluster analysis was conducted and the construction of phylogenetic tree was also performed. The results showed that structural genes including *AaF3H*, *AaLDOX* and *AaUFGT* and transcription factor genes including *AaMYB*, *AabHLH* and *AaHB2* were highly expressed at HB100, when the flesh color significantly presented red (Figure 7). In addition, the cluster analysis results suggested that *AaF3H*, *AaLDOX*, *AaUFGT, AaMYB*, *AabHLH* and *AaHB2* were clustered into one class, indicating that their expression patterns were similar to each other (Figure 8). Based on these results, a regulatory network of flavonoid biosynthesis was established to show the role of genes involved in pathways more intuitively (Figure 10). Thus, such a model of action was derived: the three transcription factors AaMYB, AabHLH and AaHB2 interact with promotors of the three structural genes *aF3H*, *AaLDOX*, and *AaUFGT* to control the expression, inducing the accumulation of metabolites and the appearance of the red phenotype of *A. arguta* fruit.

This method—combined metabolome and transcriptome—is an effective analytical method for explaining the relationship between key genes and metabolites involved in biosynthesis pathways. Using this method, we determined the candidate genes and metabolites involved in the flavonoid biosynthesis pathway, providing valuable information and a useful reference for explaining the phenomenon of red coloring of *A. arguta* fruit. Nevertheless, the specific mechanism still needs further research, and remains to be explored.

## 4. Materials and Methods

### 4.1. Fruit Materials

Two different types of kiwifruit (*Actinidia arguta*), “Hongbaoshixing” (“HB”, a kind of all-red-fleshed *A. arguta* cultivar) and “Yongfengyihao” (“YF”, a kind of all-green-fleshed *A. arguta* cultivar), were selected as experimental materials. Two different sampling stages of fruit development, defined in days after full bloom (DAFB), were 70 DAFB (70d, green fruit) and 100 DAFB (100d, the color-break stage, at which point they start to turn red). ‘YF’ fruit selected as a control case were green throughout the whole development process and were also sampled at 70d to 100d (Figure 1). Both of them are tetraploids with similar genetic backgrounds. The fruit samples at the two developmental stages described above were harvested from the National Kiwifruit Germplasm Garden of Zhengzhou Fruit Research Institute, CAAS, Henan province, China. Three biological replicates were collected per sample, each with 30 fruits randomly collected from 6 kiwifruit trees, every two of which were set as a biological replication; thus, all data were obtained based on three independent biological replicates. All flesh samples of the fresh fruits were dissected using a blade, frozen immediately in liquid nitrogen, and then stored at −80 °C until further use.

### 4.2. Metabolite Extraction and Parameter Setting

The collected samples were thawed on ice, and metabolites were extracted with 50% methanol buffer (50% solution of methanol in distilled water). Briefly, 20 μL of sample was extracted with 120 μL of precooled 50% methanol, vortexed for 1 min, and incubated at room temperature for 10 min; the extraction mixture was then stored overnight at −20 °C. After centrifugation at 4000× *g* for 20 min, the supernatants were transferred into new 96-well plates. Three independent repetitions were executed for the extraction and subsequent analysis process.

All chromatographic separations were performed using an ultra-performance liquid chromatography (UPLC) system (SCIEX, Cheshire, UK). An ACQUITY UPLC BEH Amide column (100 mm × 2.1 mm, 1.7 µm, Waters, UK) was used for the reversed phase separation. The column oven was maintained at 35 °C. The flow rate was 0.4 mL/min and the mobile phase consisted of solvent A (25 mM ammonium acetate + 25 mM NH_4_H_2_O) and solvent B (IPA:CAN = 9:1 + 0.1% formic acid). Gradient elution conditions were set as follows: 0~0.5 min, 95% B; 0.5~9.5 min, 95% to 65% B; 9.5~10.5 min, 65~40% B; 10.5~12 min, 40% B; 12~12.2 min, 40~95% B; 12.2~15 min, 95% B. The injection volume for each sample was 4 µL.

A high-resolution tandem mass spectrometer TripleTOF5600plus (SCIEX, Cheshire, UK) was used to detect metabolites eluted form the column. The Q-TOF was operated in both positive and negative ion modes. XCMS software 3.2.0 (UC, Berkeley, CA, USA) was used to control the chromatograph and mass spectrometer.

### 4.3. Identification and Quantification of Metabolite

MSConvert was used to transform LC-MS raw data into the mzXML format, which was then processed by the XCMS, CAMERA and metaX toolbox, implemented in the R software [50,51,52]. The combined retention time (RT) and m/z data were used to identify each ion.

In order to explain the physical and chemical properties and biological functions of metabolites, the online PLANTCYC (http://www.plantcyc.org/), KEGG (http://www.kegg.jp/) and in-house (http://spldatabase.saskatoonlibrary.ca/), HMDB (http://www.hmdb.ca/) databases were used to perform level-one and level-two identification and annotation. Screening and quantitative analysis for differential metabolites were conducted using metaX software (http://metax.genomics.cn/).

### 4.4. RNA Sequencing

RNA isolation, purification and monitoring, and cDNA library construction and sequencing were performed as previously described [53]. Briefly, RNA purity, concentration and integrity were checked, measured and assessed using the NanoPhotometer^®^ spectrophotometer (IMPLEN, Westlake Village, CA, USA), Qubit^®^ RNA Assay Kit in Qubit^®^ 2.0 Flurometer (Life Technologies, Carlsbad, CA, USA) and RNA Nano 6000 Assay Kit of the Agilent Bioanalyzer 2100 system (Agilent Technologies, Santa Clara, CA, USA), respectively. Sequencing libraries were generated using NEBNext^®^ Ultra™ RNA Library Prep Kit for Illumina^®^ (NEB, Ipswich, MA, USA) following manufacturer’s recommendations and were sequenced on an Illumina Hiseq platform. According to the manufacturer’s instructions, NEBNext^®^ Ultra™ RNA Library Prep Kit for Illumina^®^ (NEB, Ipswich, MA, USA) was used to generate sequencing libraries, which were then sequenced on an Illumina Hiseq platform.

### 4.5. Analysis of Transcription Factors and DEGs (Differentially Expressed Genes)

Transcription factors were predicted using iTAK software [54]. The identification and classification of transcription factors were conducted by previously described methods [55,56]. On the basic of model of negative binomial distribution, DESeq R package (1. 10. 1) was used to perform differential expression analysis by providing statistical routines used to determine differential expression by means of the digital data of gene expression. The false discovery rate was deliberately controlled by the adjusted P values, which were adjusted by Benjamini and Hochberg’s approach. The differentially expressed genes were defined as whose adjusted *p*-value < 0.05 found by DESeq.

### 4.6. RT-qPCR (Real-Time Quantitative PCR)

Total RNA was extracted using a modified CTAB (cetyltrimethyl ammonium bromide) method [16]. The testing of RNA quality and determination of RNA concentration were performed by 1.0% agarose gel electrophoresis and micro ultraviolet spectrophotometry (Thermo NanoDrop 2000, Thermo Fisher Scientific, Waltham, MA, USA), respectively. Approximately 1µg of total RNA was determined for cDNA synthesis using RevertAid™ First Strand cDNA synthesis kit (Thermo Fisher Scientific, Waltham, MA, USA). The LightCycler^®^ 480 real-time PCR system with a 96-well plate was used to conduct an amplified reaction consisting of 95 °C for 5 min, followed by 45 cycles of 10 s at 95 °C, 20 s at 60 °C, and 20 s at 72 °C in a volume of 10 μL. At the end of each experiment, a melt-curve analysis was carried out using the default parameters (5 s at 95 °C and 1 min at 65 °C). The Actinidia β-actin was used for normalization [57]. All analyses were repeated three times using biological replicates. Primer sequences are listed in Table S4.

### 4.7. Statistical Analysis

The relative expressions were calculated using the 2^−ΔΔ*C*t^ method [35], and GraphPad Prism 5 (GraphPad Software Inc., San Diego, CA, USA) was used for chart preparation. The R-3.4.2 and MEGA6 were used to conduct the heatmap and cluster analysis. IBM SPSS Statistics 20 was used to test significant differences.

## Figures and Tables

**Table 1:** Statistics of identified metabolites.

Mode	All Metabolites	All Annotated	MS2	MS1 PLANTCYC	MS1 KEGG
POS	18,598	9016	1267	3612	8070
NEG	10,239	4699	360	1968	4321
Total	28,837	13,715	1627	5580	12,391

Mode indicates that the mode of MS analysis is mainly divided into a positive ion mode and a negative ion mode; all metabolites indicates the number of substances extracted by XCMS software (3.2.0 version, UC, Berkeley, CA, USA); all annotated indicates the amount of metabolites annotated by level-one and level-two MS data; MS2 indicates the number of metabolites that could not only match that of level one m/z, but could also match that of a level-two fragment ion in the database; MS1 PLANTCYC indicates the number of metabolites that could match that of level-one m/z in the database; MS1 KEGG (Kyoto Encyclopedia of Genes and Genomes) indicates the number of metabolites assigned to KEGG pathways, POS (positive) and NEG (negative).

**Table 2:** Statistics for quantitative metabolites.

Mode	All Metabolites	High-Quality Metabolites
POS	18,598	14,132
NEG	10,239	8400
Total	28,837	22,532

Mode indicates that the mode of MS analysis is mainly divided into positive ion mode and negative ion mode; all metabolites indicates the number of substances extracted by XCMS software; high-quality metabolites indicates CV ≤ 30% of metabolites intensity in all QC samples, which were used for further analysis.

**Table 3:** Statistics for differential metabolites between different group comparisons.

Mode	Comparison	All	Up	Down
POS	HB100 vs. HB70	14,132	2421	1838
POS	YF100 vs. YF70	14,132	900	1167
POS	HB100 vs. YF100	14,132	2037	2024
POS	HB70 vs. YF70	14,132	1728	2114
NEG	HB100 vs. HB70	8400	1364	1044
NEG	YF100 vs. YF70	8400	644	859
NEG	HB100 vs. YF100	8400	1287	1118
NEG	HB70 vs. YF70	8400	1032	1119

Mode indicates that the mode of MS analysis is mainly divided into positive ion mode and negative ion mode; comparison indicates comparison between two groups, taking A vs. B as an example, B is regarded as control; all indicates all the high-quality features; up indicates that compared to B, the number of up-regulation for A; down indicates that compared to B, the number of down-regulation for A.

**Table 4:** 7 metabolites with significant difference in the comparison between HB100 and HB70.

Metabolite Name	Fold Change (HB100 vs. HB70)	KEGG Pathway
Bracteatin	4.78	Flavonoid biosynthesis
Luteolin	4.14	Flavonoid biosynthesis
Dihydromyricetin	2.10	Flavonoid biosynthesis
Cyanidin	4.01	Flavonoid biosynthesis
Pelargonidin	2.72	Flavonoid biosynthesis
Delphinidin	2.70	Flavonoid biosynthesis
(−)-epigallocatechin	2.45	Flavonoid biosynthesis

## Supplementary Materials

Supplementary materials can be found at http://www.mdpi.com/1422-0067/19/5/1471/s1.
